# Predictors of lymph node metastasis in resectable pancreatic neuroendocrine neoplasms: a single-center retrospective study

**DOI:** 10.3389/fonc.2025.1746231

**Published:** 2026-01-15

**Authors:** Xue-yang Chen, Jing Du, Juan-juan Cai, Tao Li, You-wei Chen, Lei Wang, Wei-quan Wu, Jie Dong

**Affiliations:** 1 Cancer Center, Department of Gastroenterology, Zhejiang Provincial People’s Hospital, Affiliated People’s Hospital, Hangzhou Medical College, Hangzhou, Zhejiang, China; 2 Cancer Center, Department of Pathology, Zhejiang Provincial People’s Hospital, Affiliated People’s Hospital, Hangzhou Medical College, Hangzhou, Zhejiang, China; 3 Department of Gastroenterology, Huzhou Nanxun People’s Hospital, Huzhou, Zhejiang, China; 4 Department of Clinical Medicine, Hangzhou Medical College, Hangzhou, Zhejiang, China

**Keywords:** inflammatory molecular markers, lymph node metastasis, monocyte-to-lymphocyte ratio, predictive factors, resectable pancreatic neuroendocrine neoplasms, retrospective study

## Abstract

**Background:**

Pancreatic neuroendocrine neoplasms (pNENs) present great heterogeneity in biological behavior, histological characteristics and clinical manifestations. Monocyte-to-lymphocyte ratio (MLR) is a noninvasive and easy-to-obtain indicator, that can reflect disease severity in multiple tumors. Lymph node metastasis (LNM) strongly affects the patient’s surgical approach and prognosis. Predicting LNM before surgery has significance for the guidance of clinical treatment. We aimed to evaluate the predictive factors, including MLR associated with LNM of patients with resectable pNENs in our center.

**Methods:**

A total of 64 patients who underwent pNEN resection and lymph node dissection in our hospital from July 2014 until June 2023 were included in this study. Univariate and multivariate analyses were performed to identify predictive factors for LNM by analyzing clinical data, inflammatory markers, and pathological features.

**Results:**

Among the 64 patients, 15 (23.4%) were node positive. Univariate analysis revealed that vascular invasion, peripheral nerve invasion, bilirubin level, tumor grade, tumor size and MLR (p<0.05 for all) were risk factors for LNM. Multivariate logistic analysis demonstrated that tumor size was the only independent risk factor for LNM in our study. Multivariate ROC analysis had better predictive performance than univariate analysis did.

**Conclusions:**

The preoperative MLR, vascular invasion, peripheral nerve invasion, bilirubin level, tumor grade and tumor size are potential predictors of LNM, especially during the initial diagnosis of resectable pNENs. Multivariate ROC analysis demonstrated superior performance by incorporating variables significant in univariate analysis. These factors combined can assist in clinical decision-making, such as more aggressive early intervention or intensive follow-up.

## Introduction

Pancreatic neuroendocrine neoplasms (pNENs) represent a heterogeneous group of malignancies characterized by diverse pathological features and clinical behaviors. The annual incidence is less than 1 per 100,000 individuals (1). PNENs predominantly arise sporadically; however, they may also develop in association with hereditary syndromes such as multiple endocrine neoplasia type I (MEN1) and von Hippel-Lindau (VHL) disease (2). PNENs account for approximately 2%-5% of all pancreatic neoplasms. These tumors originate from multifunctional pancreatic neuroendocrine stem cells (1, 3). The biological behavior, histological characteristics and clinical manifestations of PNENs are highly heterogeneous. These tumors can grow independently or aggressively and even metastasize at an early stage. Their biological characteristics may also change as the disease progresses (4).

Patients with G1 and G2 grade have a 10-year overall survival rate of 60-70%. Owing to the possibility of recurrence or metastasis of pNENs, the median survival of G3 patients is less than 2 years (5). Surgery is the main treatment for pNENs. The incidence of lymph node metastasis (LNM) in patients with nonfunctional pNENs with a maximum diameter ≤2 cm ranges from 17% to 27.3% (6, 7). The TNM stage of the tumor is correlated with LNM. In patients without distant metastases, tumor size and LNM strongly affect the prognosis and surgical approach (8). The routine dissection of lymph nodes increases the difficulty of surgery, prolongs the operation time and increases postoperative complications (9). To reduce patient pain and postoperative complications, it is necessary to fully evaluate LNM before surgery. Some researchers have reported that the tumor size, T stage and tumor grade are related to LNM (10).

Neutrophil-to-lymphocyte ratio (NLR), monocyte-to-lymphocyte ratio (MLR) and platelet-to-lymphocyte ratio (PLR) are noninvasive, rapid, easy-to-obtain and inexpensive indicators, that can reflect the balance of inflammation and immune activity in the body. They have been proven to be important indicators of prognosis and disease severity in a variety of tumors. These parameters have promising clinical applications (11–13).

MLR, an emerging biomarker that integrates the opposing roles of monocytes and lymphocytes, has garnered significant attention in oncology research. By quantifying the balance between these two key immune cell populations, MLR provides a dynamic reflection of the host’s immune status and its interaction with tumor biology. This biomarker has demonstrated significant predictive and prognostic value across a range of malignancies, including hepatocellular carcinoma, gastrointestinal stromal tumors, endometrial cancer, bladder cancer, and breast cancer (14). Notably, studies have shown that MLR not only serves as a baseline indicator of disease aggressiveness but also reflects changes in immune dynamics during treatment. For instance, in patients with hepatocellular carcinoma receiving locoregional therapy, elevated MLR levels at both baseline and relapse have been strongly associated with adverse outcomes, underscoring its utility in guiding therapeutic decisions and monitoring disease progression (15). As a noninvasive and cost-effective marker, MLR holds promise as a complementary tool in personalized cancer management. Further validation in large-scale prospective studies is warranted to fully establish its clinical utility across diverse cancer types and treatment modalities.

Some researchers have shown that hepatocellular carcinoma patients with a low MLR have longer recurrence-free survival (RFS) (15). MLR in adult lymphoma patients with peripheral lymph node enlargement is significantly greater than that in nonlymphoma patients. MLR is a good diagnostic marker for lymphoma (16).

When neuroendocrine cells are overactivated by chronic inflammation, they may develop atypical or neoplastic lesions (17).

NLR and PLR are independent factors that affect the prognosis of pNEN patients. A high NLR is often associated with poor prognosis (10, 13, 18, 19).

LNM is related to the prognosis of patients and influences the mode of surgery. Owing to the heterogeneity of pNENs, predicting LNM before surgery is valuable for guiding clinical treatment. Patients with the potential for LNM are screened, and lymph node dissection is recommended during tumor resection. However, there are limitations in deciding whether to perform lymph node dissection on the basis of only tumor size. More risk factors need to be revealed to comprehensively evaluate LNM to determine the appropriate timing for lymph node dissection.

Hence, we performed this retrospective study to explore the risk factors associated with LNM in patients with resectable pNENs in our center by analyzing clinical data, inflammatory markers, and pathological features.

## Materials and methods

### Study design and patients

A retrospective analysis of the clinical and pathological data of adult patients diagnosed with pNEN in our hospital from July 2014 until June 2023 was performed. Ethical approval for the study was obtained from the Ethics Committee of Zhejiang Provincial People’s Hospital(IRB No. QT2024150).

### Inclusion criteria

1. PNEN was confirmed by postoperative pathology.
2. The lesion could be treated surgically according to the preoperative evaluation.
3. The lymph nodes were dissected during the operation.

### Exclusion criteria

1. The patient underwent only biopsy, but no surgical treatment was performed at our hospital.
2. Lymph nodes were not dissected during the operation.
3. Multiple endocrine neoplasia type 1(MEN1), Von Hippel–Lindau disease, neurofibromatosis type 1(NF1), or tuberous sclerosis was diagnosed.

Data including sex, age of onset, functional status, clinical symptoms and preoperative hematological indicators, such as routine blood parameters (neutrophil count, platelet count, lymphocyte count, and monocyte count), bilirubin level, tumor markers (NSE, CEA, CA199, and AFP), tumor site, tumor size and pathological data (pathological type, pathological grade, immunohistochemical results, vascular or peripheral nerve invasion, and LNM) were collected.

### Data analysis

All the statistical analyses and graphics in this study were performed and generated by using the IBM SPSS Statistics software 20.0. Chi-square tests were used to compare detection rates between groups. The Mann–Whitney U test was used to evaluate ranked variables. Multivariate logistic regression analyses were performed to analyze risk factors for LNM. The ROC curves were used to estimate the ability of each factor to predict LNM. Cut-off values were determined using ROC curve analysis with the Youden index (maximizing [Sensitivity + Specificity - 1]). Difference with two-sided *p* values <0.05 was considered statistically significant.

## Results

### Patient characteristics, preoperative peripheral blood test results and pathological results

From July 2014 to June 2023, a total of 64 patients underwent pNEN resection and lymph node dissection in our hospital. The characteristics and results are summarized in **Table 1**. The age at diagnosis was 53.50 years (range 16-73; 47.25-62.75). Among them, 38 (59.4%) were male. Seven people had functional pNENs (10.9%) and 62 had a single NEN. Forty-three patients had no obvious discomfort at the time of visit, and 21 patients had abdominal pain, abdominal distension, poor appetite, hypoglycemia and other symptoms upon physical examination. A total of 14.1% (9/64) of patients had elevated bilirubin levels, 3.2% (2/63) had elevated CEA levels, 4.8% (3/63) had elevated CA199 levels, and 8.3% (3/36) had elevated NSE levels. The preoperative MLR was 0.185 (0.132-0.246), NLR was 1.906 (1.434-2.771) and PLR was 116.32 (94.80-159.01). The lesions of 38 patients were located in the head and neck of the pancreas, and the others were located in the tail of the body of the pancreas. The average tumor size was 3.00 cm (min 0.15cm, max 10cm; interquartile range 1.85-4.50cm). Thirty-six (56.3%) patients were diagnosed with G1 neuroendocrine tumors (NETs),21 (32.8%) with G2 NETs, 4 (6.3%) with G3 NETs, 1 (1.6%) with neuroendocrine cancer (NEC) and 1 (1.6%) with mixed neuroendocrine-non-neuroendocrine neoplasm (MiNEN). The median number of retrieved lymph nodes was 9 (interquartile range [IQR]: 2-17). We found that 76.6% (49/64) of patients were lymph node negative and that the other 23.4% (15/64) of patients were node positive. Pathology revealed that 23.4% (15/64) of the patients had vascular invasion, 23.4% (15/64) had peripheral nerve invasion, 93.5% of the patients were positive for CgA (58/62) and 100% were positive for Syn (61/61).

**Table 1:** Patient characteristics, preoperative peripheral blood test results, pathological results and univariate and multivariate analyses of clinical characteristics according to the degree of lymph node metastasis.

Factors	Total number (64)	Lymph node status		Univariate P-value	Multivariate P-value
		Negative (49)	Positive (15)		
Age	53.50 (47.25-62.75)	55.00 (47.50-63.00)	52.00 (45.00-60.00)	0.669	
Sex				0.563	
Male	38	28	10		
Female	26	21	5		
Functional status				0.545	
Functional	7	6	1		
Nonfunctional	57	43	14		
Numbers of NENs				0.427	
Single	62	47	15		
Multiple	2	2	0		
Symptoms				0.755	
Yes	21	17	4		
No	43	32	11		
Tumor size (cm)	3.00 (1.85-4.50)	2.50 (1.70-4.00)	4.50 (3.00-7.00)	0.013	0.033
Tumor location				0.563	
Head and neck	38	28	10		
Body and tail	26	21	5		
Tumor grade				0.017	>0.05
G1	36	32	4		
G2	21	12	9		
G3	4	3	1		
NEC	2	1	1		
MiNEN	1	1	0		
Vascular invasion				<0.05	0.755
Yes	15	6	9		
No	49	43	6		
Peripheral nerve invasion				0.015	0.250
Yes	15	8	7		
No	49	41	8		
CgA				0.969	
Positive	58	44	14		
Negative	4	3	1		
Syn				-	
Positive	61	46	15		
Negative	0	0	0		
Bilirubin level	64			0.027	0.484
Normal	55	45	10		
Abnormal	9	4	5		
CEA	63			0.442	
Normal	61	47	14		
Abnormal	2	2	0		
CA199	63			0.058	
Normal	60	48	12		
Abnormal	3	1	2		
NSE	36				
Normal	33	25	8		
Abnormal	3	2	1		
MLR	0.185 (0.132-0.246)	0.179 (0.128-0.225)	0.200 (0.167-0.500)	0.040	0.164
NLR	1.906 (1.434-2.771)	1.800 (1.413-2.740)	2.041 (1.583-3.875)	0.425	
PLR	116.32 (94.80-159.01)	115.663 (91.862-151.379)	130.833 (96.250-180.233)	0.264	

### Relationships between LN metastasis and MLR and other clinicopathologic characteristics

The χ^2^ test revealed significant differences in vascular invasion(P<0.05), peripheral nerve invasion (P=0.015), bilirubin level(P=0.027) between patients with and without LNM. Tumor grade (P = 0.017), tumor size (P=0.013), MLR (P=0.040) in patients with LNM were significantly greater in patients with LNM than in patients without LNM. These six variables were selected as potential independent risk factors for the ROC curve and multivariate analyses. Multivariate logistic regression indicated that tumor size(P=0.033) was the only independent risk factor for LNM (**Table 1**).

**Figure 1:**
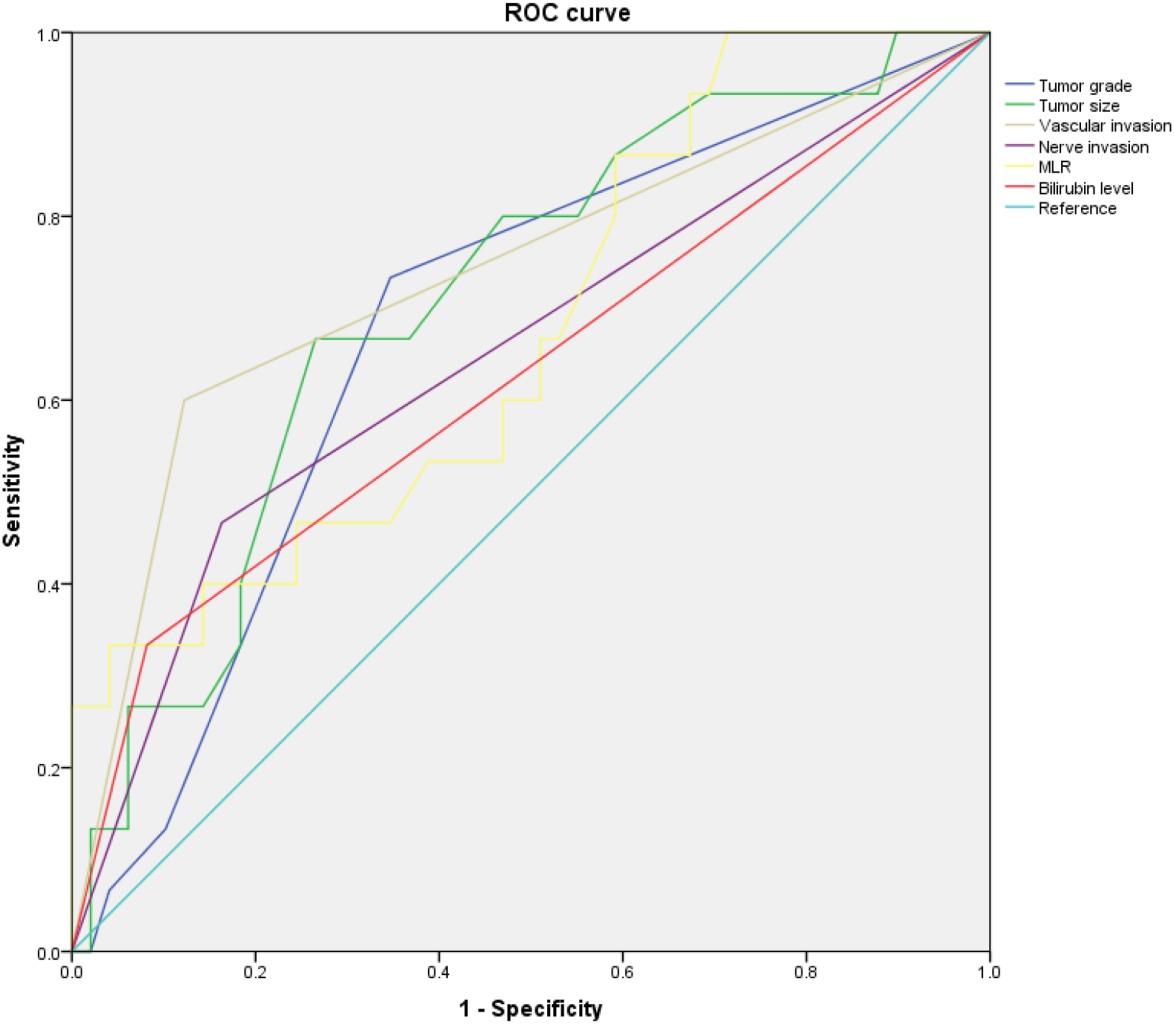
The ROC curves of the six variables.

The ROC curve analysis for single-factor prediction of LNM revealed that the NEN grade had an AUC of 0.679 (95% CI [0.528, 0.830], P = 0.037), tumor size had an AUC of 0.710(95% CI [0.564,0.857], P = 0.014), MLR had an AUC of 0.676(95% CI [0.521,0.830], P = 0.041), vascular invasion had an AUC of 0.739(P=0.005)(**Figure 1**, **Table 2**). These variables predict LNM. The AUC of the combination of the four significant factors(0.898, 95% CI [0.815,0.981], P<0.05) was greater than the AUC of any individual factor.

**Table 2:** ROC curve analysis for the prediction of LNM.

Factors	ACU	P-value	95% CI
Tumor grade	0.679	0.037	0.528-0.830
Tumor size	0.710	0.014	0.564-0.857
Vascular invasion	0.739	0.005	0.578-0.899
Nerve invasion	0.652	0.077	0.481-0.822
MLR	0.676	0.041	0.521-0.830
Bilirubin level	0.626	0.143	0.450-0.802

## Discussion

The incidence of pNENs is low, ranging from 0.1/100,000 to 0.48/100,000 (20). However, with improvements in diagnostic equipment, pathological classification systems, and physician knowledge, the incidence of pNENs in Europe, America, Japan, and China is increasing yearly (21), and an increasing number of studies have investigated the factors affecting the prognosis of pNENs. A previous literature review revealed that age, sex, race, tumor size, tumor marker levels, histological pathology, LNM and tumor stage are associated with the prognosis of pNENs. At present, LNM is considered one of the most important factors for poor prognosis (8, 22). Multiple studies based on the SEER database have shown that tumor location (pancreatic head), tumor grade, tumor size, T stage, Ki-67, vascular invasion and nerve invasion are associated with LNM. Patients with a higher risk of LNM have a worse prognosis (23, 24). Our study also revealed that tumor grade, tumor size, vascular invasion, nerve invasion, and bilirubin level were associated with LNM, and that tumor size was an independent risk factor for LNM, which was consistent with the results of previous studies based on databases. In our study, the level of bilirubin was associated with LNM. We speculate that tumors located in the head of the pancreas are more likely to compress the bile duct and cause increased bilirubin levels.

According to the National Cancer Database (NCDB) (2004-2014), in a retrospective study of 2664 patients with pNENs, 2132 patients underwent lymph node dissection, 28% of whom had lymph node metastases, and the overall survival time of patients with and without lymph node dissection was 152.8 and 147.3 months, respectively (p = 0.61) (25). Krampitz et al. reported no significant difference in 10-year overall survival between patients with and without LNM (26), however, neither study suggested that lymph node dissection should be routinely avoided in all pNEN patients. Obtaining an adequate and consistent nodal yield during surgery and pathology is essential for accurate staging.

Although well-differentiated, low-grade pNENs are often considered relatively indolent tumors, patients with LNM have a recurrence rate as high as 40% to 50% (27). The recurrence rate of patients with liver metastases after surgery is as high as 95% (28). Therefore, we should be cautious about detecting LNM.

Inflammatory molecular markers have been widely used in the diagnosis and prognosis of various tumors. Long-term inflammation can promote tumorigenesis, and various cells in the tumor microenvironment can also secrete a variety of inflammatory factors. The systemic inflammatory response is closely related to the occurrence, development and metastasis of tumors (29).

Lymphocyte infiltration within the tumor microenvironment serves as a prognostic indicator of robust antitumor immune responses. Accumulating evidence has demonstrated that tumors with dense lymphocyte infiltration are often associated with heightened immune surveillance and more effective immune-mediated tumor control. Low lymphocyte counts are associated with adverse clinical outcomes, suggesting a weakened immune response in the host. This may be explained by reduced numbers of CD4+ T cells, which are vital for tumor suppression and defence (30). Elevated lymphocyte counts in peripheral blood or tumor tissue are consistently correlated with improved overall survival and better clinical outcomes in patients with various malignancies. This relationship highlights the critical role of lymphocytes in orchestrating adaptive immune responses against cancer and their potential as biomarkers for stratifying patients on the basis of prognosis (13).

Monocytes, key components of the mononuclear phagocyte system during the innate immune response, have emerged as critical regulators in cancer initiation and progression (31). These cells exert immunosuppressive effects through the production of reactive oxygen species (ROS), reactive nitrogen species (RNS), and the secretion of key regulatory cytokines, such as interleukin-10 (IL-10), indoleamine 2,3-dioxygenase (IDO), and transforming growth factor-β (TGF-β), which collectively disrupt T-cell metabolism and function (32). By targeting critical metabolic pathways in T-cells, these signals not only suppress T-cell proliferation and activation but also induce a state of T-cell exhaustion or dysfunction, thereby undermining antitumor immunity. The accumulation of monocyte-derived macrophages and their immunosuppressive activity within the tumor microenvironment thus represent critical drivers of immune evasion and disease progression, highlighting their potential as therapeutic targets for restoring effective antitumor immune responses (33).

Increased monocyte numbers and decreased lymphocyte numbers in inflammatory diseases lead to an imbalanced MLR. Some studies have also shown that pNEN patients with a low MLR at baseline have a better prognosis, and that their MLR is an independent predictor of tumor recurrence (34, 35). Few studies have investigated the relationships between NLR, PLR, MLR and LNM. A study involving 95 patients with resectable pNENs revealed that RFS was greater in patients without LNM. The preoperative NLR level was an independent risk factor for LNM. A higher the NLR resulted in a higher the risk of LNM. PLR and MLR were not included (10). In our study, NLR and PLR were not associated with LNM, whereas MLR was a risk factor for LNM and had a predictive effect on LNM. The ability to predict the risk of LNM from tumor size alone was weaker than that of the multivariate combination.

The depletion of intratumoral lymphocytes compromises immune surveillance, directly contributing to enhanced tumor progression, recurrence, and metastasis. Conversely, monocyte-derived tumor-associated macrophages (TAMs) play a critical role in oncogenesis by promoting tumor invasion, angiogenesis, and immunosuppression, primarily through their polarization into M2 phenotype. A high MLR may be closely associated with postoperative recurrence. Therefore, MLR may help optimize treatment strategies, such as more aggressive early intervention or intensive follow-up (34).

The combination of the four significant factors—NEN grade, tumor size, monocyte-to-lymphocyte ratio (MLR), and vascular invasion—demonstrated superior predictive performance compared with any individual factor alone. Specifically, the AUC of the combined model was 0.898 (95% CI 0.815–0.981, *P* < 0.05), indicating that the integration of these factors significantly enhanced the ability of the model to discriminate between outcomes. While models with moderate AUC values may exhibit limited standalone predictive power, their clinical utility can be significantly amplified when they are integrated with established clinical indicators such as imaging findings or biomarkers. The results highlight the value of multimodal approaches in refining risk stratification and guiding personalized patient management.

Our study had several limitations. First, some selection biases may exist since our study was a retrospective single-center study. In the future, a multicenter study could be performed to verify our findings. Second, because of the low incidence of pNEN, the number of samples included in our study that met the inclusion criteria was limited. Lymph node dissection extent was based on surgeon judgment and preoperative assessment.

## Conclusion

The preoperative MLR, vascular invasion, peripheral nerve invasion, bilirubin level, tumor grade and tumor size are potential predictors of LNM, especially during the initial diagnosis of resectable pNENs. In our study, tumor size was the only independent risk factor for LNM. Multivariate ROC analysis demonstrated superior performance by incorporating variables significant in univariate analysis. Thus, taking the above factors into consideration can help optimize treatment strategies, such as more aggressive early intervention or intensive follow-up.

## Data Availability

The original contributions presented in the study are included in the article/supplementary material. Further inquiries can be directed to the corresponding authors.
